# A methodological framework for drug development in rare diseases

**DOI:** 10.1186/s13023-014-0164-y

**Published:** 2014-11-18

**Authors:** Patrice Nony, Polina Kurbatova, Agathe Bajard, Salma Malik, Charlotte Castellan, Sylvie Chabaud, Vitaly Volpert, Nathalie Eymard, Behrouz Kassai, Catherine Cornu, The CRESim and Epi-CRESim study groups

**Affiliations:** CHU Lyon, Service de Pharmacologie Clinique et Essais Thérapeutiques, Lyon, France; University of Lyon 1, UMR 5558, CNRS, Lyon, France; Hôpital Louis Pradel, Centre d’Investigation Clinique, INSERM CIC1407/UMR5558, Bron, France; Unité de Biostatistique et d’Evaluation des Thérapeutiques, Centre Léon Bérard, Lyon, France; Institut Camille Jordan UMR 5208 Université Claude Bernard, Lyon 1, France; Service de Pharmacologie Clinique et Essais Thérapeutiques-HCL, Groupement Hospitalier Est, Hôpital Cardiovasculaire et Pneumologique Louis Pradel, 28, Avenue du Doyen Lépine, 69677 Bron Cedex, France

**Keywords:** Rare diseases, Drug development, Integrative modeling, Clinical trial simulation

## Abstract

**Introduction:**

Developing orphan drugs is challenging because of their severity and the requisite for effective drugs. The small number of patients does not allow conducting adequately powered randomized controlled trials (RCTs). There is a need to develop high quality, ethically investigated, and appropriately authorized medicines, without subjecting patients to unnecessary trials.

**Aims and Objectives:**

The main aim is to develop generalizable framework for choosing the best-performing drug/endpoint/design combinations in orphan drug development using an in silico modeling and trial simulation approach. The two main objectives were (i) to provide a global strategy for each disease to identify the most relevant drugs to be evaluated in specific patients during phase III RCTs, (ii) and select the best design for each drug to be used in future RCTs.

**Methodological approach:**

In silico phase III RCT simulation will be used to find the optimal trial design and was carried out in two steps: (i) statistical analysis of available clinical databases and (ii) integrative modeling that combines mathematical models for diseases with pharmacokinetic-pharmacodynamics models for the selected drug candidates.

**Conclusion:**

There is a need to speed up the process of orphan drug development, develop new methods for translational research and personalized medicine, and contribute to European Medicines Agency guidelines. The approach presented here offers many perspectives in clinical trial conception.

## Introduction

The European Commission on Public Health defines rare diseases as “life-threatening or chronically debilitating diseases which are of such a low prevalence that special combined efforts are needed to address them” [1]. The term low prevalence is defined as less than 1 in 2,000 people affected. It has been estimated that there are between 6,000 and 8,000 rare diseases that may affect up to 30 million people in the European Union alone. About 80% of these rare diseases have an identified genetic origin involving one or several genes or chromosomal abnormalities [2]. The others are caused by infections (bacterial or viral), or allergies, or are due to degenerative, proliferative or teratogenic (chemicals, radiations, etc.) causes. Some rare diseases are also caused by a combination of genetic and environmental factors [2]. Rare diseases include a wide range of disorders and symptoms across diseases and patients suffering from the same disease. Therefore, it is impossible to develop public health policies specific to each rare disease. A global approach to rare diseases is required to create policies on scientific and biomedical research, drug research and development, industry policies, training, social benefits, hospitalization, and outpatient treatment.

Orphan drugs are developed to treat rare diseases, often known as orphan diseases (Table 1). Orphan drugs follow the same regulatory development path as other pharmaceutical products, with studies focusing on pharmacokinetics, pharmacodynamics, dosing, stability, safety, and efficacy. Adequately powered randomized controlled trials (RCTs) may be difficult to conduct due to the small number of potential participants. Drug manufacturers and regulatory agencies have traditionally been skeptical of small clinical trials, mainly because of their low statistical power and lack of transposability. New approaches to protocol design are currently required for trials with small sample sizes that can assess the potential therapeutic efficacy of drugs, biological products, medical devices, and other medical interventions. We propose here a strategy in order to optimize the clinical drug development (designing of phase III trials) in the field of rare diseases.

**Table 1:** **Orphan and non-orphan medicine (adapted from Spilker B)**

**Orphan medicine**	**Non-orphan medicine**
Used in a limited patient population	Used in a large patient population
Often used by only a few specialists	Generally used by a wide variety or number of physicians
The manufacturer often loses money	The manufacturer is more likely to make money
May require less patient exposure to obtain marketing authorization	Usually requires a standard quantity of data before marketing authorization

### The standard approach to clinical drug evaluation in humans

When evaluating the intended effects of drugs, well-conducted RCTs have been widely accepted as the scientific standard [3]. Randomization is the key component of RCTs. It allows focusing only on the outcome variable(s) in different treatment groups when assessing an unbiased treatment effect. As proper randomization confirms that the treatment groups differ on all known and unknown prognostic factors only by chance, probability theory can be used to make interpretations about the treatment effect in the population under study (confidence intervals, significance, etc.) and it removes potential selection bias [4].

Randomization does not ensure equality for all prognostic factors in the treatment groups, especially with small sample sizes, but it does ensure that confidence intervals and p-values are validated using probability theory [5]. Occasionally randomized comparison of treatments may not be considered feasible due to ethical, economical and other limitations related to the rareness of the disease [6]. RCTs usually exclude particular groups of patients (because of age, other drug usage, or noncompliance). They are mainly conducted under strict, protocol-driven conditions and experimental drugs are generally taken for shorter periods than drugs used in clinical practice. The main alternatives are observational studies. Their validity for assessing intended effects of therapies has long been debated and remains controversial [7-9].

### Limitations of using the standard approach in rare diseases

The small numbers of patients, who are spread out over a wide geographical area make it difficult to carry out traditional clinical trials (i.e. RCTs in parallel groups) with enough power. There is a need for individually-tailored therapies and the inclusion of specific populations. It is also a useful approach to favor investigators who are specialized in the disease, use patient inclusion criteria that are as inclusive as possible, measure the treatment effect with an endpoint that is as standard as possible, and include patient associations and public institutions when preparing the trial. For the same disease, there may be several new treatments to evaluate. The total number of clinical trials is limited, so a choice must be made to favor the evaluation of one treatment over others. If we want to evaluate a treatment strategy while carrying out complementary paraclinical evaluation (i.e. a theranostic approach), we have to choose one or a few limited strategies to compare to the conventional strategy because of the large number of possible strategies.

Unfortunately, the results of clinical trials that are most often published have a low level of evidence. Studies without a control group that are carried out according to a “before/after” methodology and/or use of historical comparisons are not appropriate for drug evaluation because they are potentially biased [10-13]. The choice of study design for small clinical trials may be based on the kind of endpoint and follow-up duration [14,15], but this type of approach does not allow to take into account the trial’s aims, the number of patients required to have enough statistical power, intra- and inter-subject variability, and the duration and cost of the trial (according to patients, investigators, and the sponsor) [16]. With this in mind, we propose a two-stage approach, that includes (i) collecting and retrospectively analyzing available epidemiological and RCT databases and (ii) using an in silico modeling and simulation approach.

### Methodological approach

**Figure 1:**
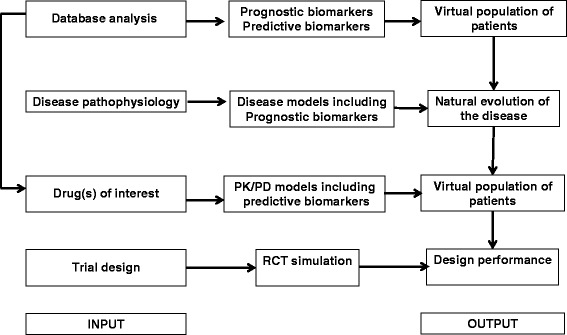
**Flow chart for modeling and simulation approach.**

**Figure 2:**
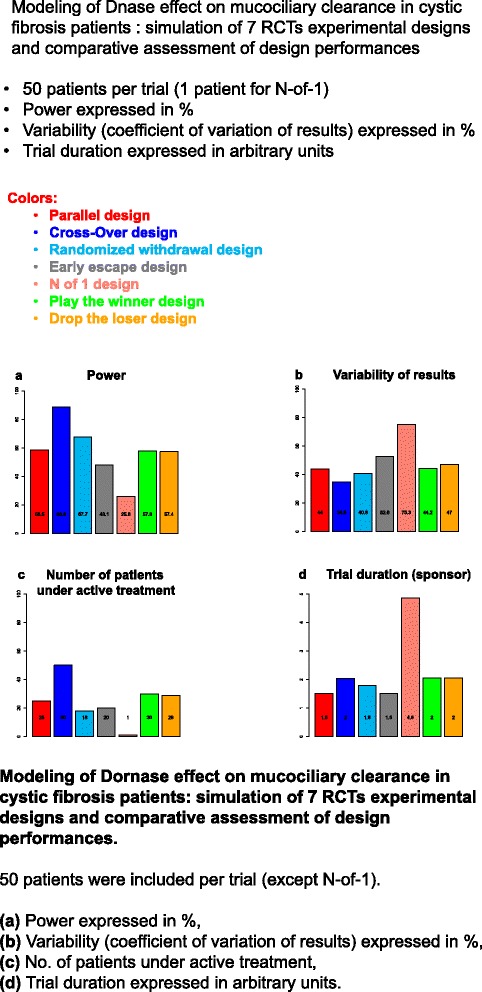
**Modeling of Dornase effect on mucociliary clearance in cystic fibrosis patients: simulation of 7 RCTs experimental designs and comparative assessment of design performances.** 50 patients were included per trial (except N-of-1). **(a)** Power expressed in %, **(b)** Variability (coefficient of variation of results) expressed in %, **(c)** No. of patients under active treatment, **(d)** Trial duration expressed in arbitrary units.

The methodological approach used here comprised of several steps (Table 2). For a given rare disease, the aim is to help identify the treatment that seems the most efficacious out of several potential treatments and further tested in a phase III clinical trial with an optimal experimental design in patients chosen where necessary based on specific prognostic and predictive markers. This process is done by retrospectively analyzing all available clinical databases and creating in silico (mathematical) models describing the disease, each treatment effect, and the results of clinical trials simulated in different patient populations and according to different study designs (Figure 1). The results are then ranked, according to potential efficacy, adverse events, number of patients needed, and the cost and duration of trials (Figure 2). This will allow selection of the drug(s) among old compounds to be further evaluated in a phase III trial using the most appropriate study design.

**Table 2:** **Steps involved in methodological approach**

**Steps**	**Approaches**	**Methods**
**Step 1**	**Use available knowledge**	**Bibliography**
		**(i)** Pathophysiology, diagnosis, therapeutics, pharmacology
		**(ii)**Discursive and mathematical models for the disease
		**(iii)**Discursive and mathematical models for the drug effect(s)
		**Individual epidemiological and RCT databases**
		**(i)** Statistical approaches for analysis
		**(ii)** Identify prognostic biomarkers
		**(iii)** Identify N potential drugs (or therapeutic strategies) for evaluation in phase III RCTs
		**(iv)** Identify predictive biomarkers for these N drugs (e.g. interactions between patient characteristics and drug efficacy)
		**(v)** Validate drug-disease models
**Step 2**	**Drug-disease modeling for the N treatments identified above**	**Treatment 1:** (Disease model + Drug effect model 1)
		**Treatment i:** (Disease model + Drug effect model i)
		**Treatment N:** (Disease model + Drug effect model N)
**Step 3**	**Drug-disease modeling for the N treatments above in patients whose characteristics may interact with drug efficacy**	**Treatment 1:** (Disease model + Drug effect model 1) in patients whose specific characteristics interact with treatment effect 1
		**Treatment i:** (Disease model + Drug effect model i) in patients whose specific characteristics interact with treatment effect i
		**Treatment N:** (Disease model + Drug effect model N) in patients whose specific characteristics interact with treatment effect N
**Step 4**	**Experimental RCT design modeling (including orthogonal approaches) for N conditions above**	P experimental designs *N situations
**Step 5**	Simulate these N*P options	Results ordered in terms of potential efficacy, adverse events, number of needed patients, cost (including trial duration)
**Step 6**	**Identify the most relevant drugs** to be evaluated in phase III RCTs and the RCT design to be used for each of them	Multiple-criteria decision analysis approaches

### Collection of databases

The first step is to collect data that are as exhaustive as possible on a given rare disease from existing clinical databases [17]. The main items used to characterize a database are listed in Table 3. For epidemiological studies, the highest level of evidence is in patient registries that are on-going, exhaustive systems of data collection of patients with the same disease(s) from a geographically-defined population over an extended period of time. Patient data registries constitute key instruments supporting health service planning, increasing knowledge on rare diseases, and support research by pooling data. The objectives of these databases are to include extensive information on all forms of a given rare disease grouped within several main categories. Long-term objectives include improving disease management and patient care, targeting preventive measures to lower co-morbidities, thereby improving the quality of life of patients. Such epidemiological registries are a basic prerequisite for obtaining a comprehensive and accurate description of a rare disease. There is no formal method to conduct an exhaustive search for such available databases. As in a meta-analytic process, such a search should be performed in computerized and non-computerized databases. Bibliographies of relevant papers and conference proceedings have also to be hand searched and experts, drug manufacturers, and primary authors must be contacted for information on additional existing datasets. The search must not be limited by language. Contacts with patient associations are also always necessary. In the field of cystic fibrosis (CF), an example of such overview of international literature from CF registries is given by Salvatore et al. [18-20] and Buzzetti et al. [21].

**Table 3:** **Items to be considered for a standardized description of databases/registries**

**Items**	**Examples**
Type of database	Registry
Objectives	Include extensive information on all forms of a given rare disease grouped within several main categories
Database conception	Team composed of clinicians and databank professionals
Recruitment sources	Nationwide recruited patients
Inclusion/exclusion criteria	Number of variables collected per patient at each visit
Follow-up characteristics:	Number of follow-up visits per year for each patient
Main results	Launch date
	Total number of patients
	Median duration of follow-up
	Number of centers
Database perpetuation	Specific contacts with the coordinating team through e-mail, phone calls and local visits, periodic meetings with all affiliated centers.
Technical aspects	Use of a secured Internet protocol into a safe database through a web interface and specification of the characteristics of the available export formats
Ergonomic aspects	Rolling menus and data entry forms accessible to unskilled users
Cross-linking of registries	In order to share and compare data with other similar registries in other nations
Quality control	A data manager/technical team should be in charge of quality control, monitoring for data coherence, absence of duplicates, and transfer of data
Organization/management rules	A charter describes general rules relating to organization and rules governing access to data
Data sharing	Rules for sharing: for cross-centre studies, the respective centers must agree explicitly to share its anonymous data with other centers
Confidentiality of patient records	All subjects receive a unique study-identification code, which anonymizes the records. Only the registry's main investigators know the code and are able to link an individual report to an individual patient
Ethical considerations	Informed consent characteristics
Funding sources, competing interests	To be extensively specified

### Analysis of available individual databases

#### Retrospective identification and validation of prognostic biomarkers

A biomarker is considered prognostic when there is an association between the marker's values at baseline or changes in the marker over time and a clinical endpoint, separate from treatment. To be validated, its association with the clinical endpoint should be repeatedly demonstrated in independent studies and preferably across a range of clinical situations. Contrary to common belief, heterogeneity is more often an asset than a liability from a statistical point of view. Retrospective studies may be sufficient for the initial identification and statistical validation of prognostic biomarkers, but the biomarker’s clinical utility may need to be confirmed in prospective studies [22].

#### Retrospective identification of predictive biomarkers

A biomarker is considered predictive if the baseline value or changes in value over time are shown to predict the efficacy or toxicity of a treatment when assessed by a defined clinical endpoint. For a putative predictive biomarker to be validated, its ability to predict the effects of treatment should be repeatedly demonstrated in multiple studies. The statistical identification of predictive markers requires data from RCTs that include patients with high and low levels of the biomarker. Retrospective analyses may be sufficient to identify candidate predictive biomarkers and validate them well enough to be incorporated into trial designs and clinical practice. But prospective clinical trials may still be needed for definitive evidence [22]. However, identification of prognostic and predictive biomarkers with an adequate power requires datasets including a sufficient number of patients. This may be not possible in ‘very rare’ or ‘ultra-rare’ diseases, i.e., diseases affecting fewer than 20 patients per million of population (or, one patient per 50,000 people) and most ultra-rare diseases affect far fewer than this as few as one per million or less.

#### Retrospective identification of potential treatments of interest

Many different methods have been proposed to assess treatment effects in observational studies [23]. With all these methods, the main objective is to deal with the potential bias caused by the nonrandomized assignment of treatments, i.e. confounding [24]. The most frequently used methods are (i) *Observational study designs* (historical controls [25], candidates for treatment, comparing treatments for the same indication, case-crossover and case-time-control design [26-29], (ii) *Data-analytical techniques* (stratification and matching on certain covariates [30], asymmetric stratification [31], common multivariable statistical techniques (multivariable linear regression, logistic regression, and cox proportional hazards regression) [32,33] and propensity score adjustment [34,35], multivariate confounder score [36], instrumental variables [37], simultaneous equations and two-stage least squares [38].

### Disease/drug effect modeling and RCT simulation

#### Modeling and simulation

A model is the simplified representation of a process or a system using physical or information technology methods, logical relationships, or mathematical formulas. A model generally corresponds to any coherent construction based on a definite collection of observations and experimental facts, in short knowledge, about the studied phenomenon. A simulation predicts output functions by changing the inputs using a logical and/or mathematical model of the studied process.

The main objectives of modeling/simulation have traditionally been to: describe/explain, summarize, predict, and teach. Another objective of modeling/simulation is to identify the “key points” of the disease mechanism, identify the characteristics of patients who respond to treatment, or even propose biomarkers as potential endpoints [39,40].

The following two approaches are opposed to each other in modeling, but they are in fact complementary (i) using real data to determine the structure and parameters of the experimental data model (minimization techniques), (ii) using different models that have already been published in the scientific literature and putting them together (the “Lego®-like principle”). Two model types are traditionally adapted; the phenomenological/empirical model and the mechanistic model involves collecting and critically analyzing available knowledge on the studied problem, choosing biological hypotheses (simplified hypotheses or ones that replace insufficient knowledge), designing and writing the discursive model, setting up equations, determining model parameters, implementing analyzing the model, studying its robustness, and carrying out simulations. During these steps, the principles of parsimony and reality should always be respected.

#### Disease/drug effect modeling

So far, many disease models have been published in the literature. Their mathematical formulation is mainly based on ordinary differential equations (ODEs) and/or partial differential equations (PDEs) and these models often allow to simulate biomarker evolution during disease development [41-43]. Treatment effect modeling is most often based on pharmacokinetic-pharmacodynamics relationships and models on this topic are already available [44]. These models are especially useful for predicting biomarker changes after changing the dosage of an administered treatment [45,46]. In cystic fibrosis, Smith et al. [47] reviewed existing mathematical models of the fluid mechanics of mucociliary clearance, taking into account the morphology of the bronchial and tracheal airway surface liquid and ciliated epithelium, the cilia beat cycle, beat frequency and metachronal coordination and also the rheology of the mucous layer. For Dornase alpha, two sub models were considered: its effect on sputum viscosity described by Shak et al. [48] and its differential deposition after inhalation by Yeh et al. [49].

### Randomized controlled trial (RCT) modeling

#### Potential experimental designs

Because observational studies are not valid alternatives to RCTs, specific experimental designs for RCTs have been developed in addition to the parallel-group design (in which participants are randomized to one of two or more arms, active control(s), or a placebo) [14,50]. Each design has its own characteristics and objectives and not all the designs listed below can be proposed in a given [drug, disease, endpoint] context [11]. These include crossover, factorial, randomized withdrawal, and early-escape designs. The crossover design compares two or more treatments by randomly assigning each participant to receive study treatments in a different sequence. Once participants finish a treatment, they are switched to another one. With the factorial design, two or more treatments are evaluated simultaneously with the same participant population using randomization with various treatment combinations.

With the randomized withdrawal design, participants who respond positively to a study treatment are randomized to continue receiving that treatment or receive a placebo. The early-escape design is another way to minimize participants’ duration of exposure to a placebo by removing them from the study if they do not respond to a defined extent.

Single-subject (N-of-1), sequential, and adaptive designs have been developed for small-size studies. The N-of-1 trial design is a randomized multi-crossover study of an individual patient’s responses to a set of treatments (usually two). Treatments are randomly assigned individually or within paired periods and given applied to the patient. The patient’s disease status is measured at set time intervals, corresponding to different treatment periods. After several crossover periods, comparisons are made between the outcomes obtained for each drug.

The sequential design was created because the single-stage design can be difficult to implement due to ethical problems (impossibility of stopping an ongoing trial even if the early data show a clear difference between treatments) and/or economic reasons (the sample size is sometimes very large). With early termination procedures, repeated statistical analyses can be performed throughout the trial recruitment period and stop the trial as soon as you have enough data, while maintaining a pre-specified alpha level.

In adaptive design assignment probabilities are skewed to favor the best-performing treatment in ongoing trials. The “play-the-winner” rule is the major advantage of the adaptive design because more patients will be assigned to the more successful treatment over time. In other possible designs (randomized placebo phase, stepped wedge trials) either the time spent on placebo is minimized or all patients receive the active treatment at the end of the trial. This is very important when studying treatments for life-threatening rare diseases, especially with the ethical issues involved (i.e. the need to minimize placebo administration in severe patients).

#### Orthogonal approaches

In addition to the above mentioned designs, “orthogonal” or “meta” methods may also be used. With the Bayesian approach, researchers adapt the trial through information collected during the trial. This makes it possible to run smaller more informative trials and patients receive better treatment. Collected results can be assessed at any time, with the possibility of modifying the trial design e.g. one may slow, stop, or expand accrual; imbalance randomization to favor better-performing therapies; drop or add treatment arms; or change the trial population to focus on patient subsets that respond better to study treatments. *Multi-stage designs and the meta-analytic approach should also be considered*.

### Randomized controlled trial (RCT) simulation

Simulation of in silico trials requires mathematical models of the disease, drug/patient interaction, and the experimental design [51]. These models often exist already and are published, but they usually address a specific aspect of the problem, e.g. pharmacokinetic (PK) and pharmacokinetics/pharmacodynamics (PK/PD) models in adults for a given drug, pathophysiology, and disease development (such as receptor function, biomarker action, and genetic aspects). Published models may be found in bibliographic databases. But additional models may have to be developed if necessary.

A simulation plan is then set-up with a list of linked models [45,52]. For a given therapeutic strategy, each simulation model is classified by one of these sub-models:

(i) The input-output (IO) sub-model predicts patient outcomes. It includes a pathophysiological model of the disease (if any) and PK/PD drug properties [53]. The model structure and parameters must be based on existing data from clinical studies to adequately simulate drug (and metabolite) concentrations, biomarkers of therapeutic or toxicological response, or the incidence of a clinical outcome or adverse event.

(ii) The covariate distribution sub-model describes patient characteristics and is created using existing patient databases;

(iii) The execution sub-model describes the characteristics of experimental designs and protocol deviations (i.e. either patient-related or investigator-related) [52]. All protocol deviations are unexpected by nature and only probabilistic models can be used to simulate them [46]. The full model will express the quantitative therapeutic effect as the sum of the IO sub-model and execution sub-model [39,40].

The simulation process is divided into two steps: the simulation of a virtual population of patients, and the simulation of RCTs using specific experimental designs. Simulation of a virtual population of N virtual patients is generated and several covariate values are randomly assigned to each patient: IO sub-model parameters, for therapeutic and adverse effects, execution sub-model parameters, and covariates for investigators (center) or patients (inclusion/exclusion criteria, baseline characteristics including prognosis and predictive biomarkers). Samples of patients are then randomly drawn from this population for inclusion in each clinical trial.

In simulation of RCTs random samples of patients drawn from the virtual population are included in silico clinical trials. Random treatment allocation is based on a series of random-permutation blocks in order to avoid an imbalance between the treated and control groups for each trial when appropriate. The diversity of drug-patient interactions for therapeutic and adverse effects is simulated using the variance of each parameter distribution of the IO sub-model. Some protocol deviations may be added to the full model. These are either treatment-related (e.g. switching to another treatment) or patient-related (e.g. a missed appointment or definitive dropout). For a given disease, a given number of virtual trials is independently simulated for each experimental design.

### Analysis of simulation results

#### Statistical analysis of the results of each RCT

Depending on each study design, different statistical methods may be used such as parametric and/or non-parametric tests, hierarchical models, and/or sequential analysis (e.g. using a triangular test).

#### Analysis of final results

The final analysis should determine the most relevant drugs (multiple-criteria decision analysis approaches) and experimental designs to be evaluated in phase III RCTs. This analysis would be mostly descriptive. Each situation, (i.e. trial design and “rare disease-drug” pair) should be ranked according to the number of times significant result is attained in each trial. This final hierarchy takes into account the precision of treatment effect estimations and trial duration. Figure 2 shows what could be a graphical representation of the main results of our approach taking the effect of Dornase alpha in CF patients as an example and using mucociliary clearance as the main endpoint. According to the trial lists preference, either a high precision or a high power or a reduced time for patients, investigators or sponsor can be favored for choosing the most appropriate design.

### European Child-Rare-Euro-Simulation (CRESim) Project

The main objective of this ongoing Child-Rare-Euro-Simulation (CRESim) project is to create a platform (using cloud computing technology) for performing in silico experiments that assess RCT designs for drug evaluation in children with rare diseases. This project is funded by the European Union’s ERA-Net PrioMedChild (Priority Medicines for Children). For demonstration purposes, three diseases are studied: Dravet Syndrome (DS) causing severe myoclonic epilepsy in infancy, cystic fibrosis (CF), and lymphoblastic lymphoma (LL). An example of preliminary results in CF has been shown in Figure 2.

### Future directions

The approach proposed above may be applied to several different situations by combining three aspects diseases-drugs-endpoints and could be integrated into a translational research process. Existing databases could be analyzed to identify prognostic and predictive biomarkers, potential treatments, and for the digital validation of models. This approach could be implemented in a larger perspective combining different diagnostic strategies. Lastly, this approach could contribute to the development of European Medicines Agency guidelines.

## Conclusion

We anticipate this approach will be useful for the greater orphan disease research community and provide funding organizations and patient advocacy groups with suggestions for the best way forward. In addition to enabling academic clinical research, strategies such as this may also help start-up companies obtain funding, as well as increase the pharmaceutical industry’s commitment to orphan drug development. However, this proposed in silico approach for rare diseases would need a final and consistent validation of the treatment effect using always an in vivo clinical trial carried out in real patients. Our approach could be considered as a potential way to foster reflection on orphan drug development.

## Abbreviations

CRESim: Child rare euro simulations
CF: Cystic fibrosis
DV: Dravet syndrome
IO: Input Output
LL: Lymphoblastic lymphoma
RCTs: Randomized controlled trials
ODEs: Ordinary differential equations
PDEs: Partial differential equations
PK/PD: Pharmacokinetic/pharmacodynamic
